# Assessing upper motor neuron dysfunction in ALS: from TMS-EEG and EMG neurophysiology to a combined tFUS-TMS translational framework

**DOI:** 10.3389/fneur.2026.1798525

**Published:** 2026-06-11

**Authors:** Ahmadreza Keihani, Mahsa Hassani, Seyed Saman Sajadi, Seyedeh Atena Modarresi, Marziyeh Khoshkholgh, Mahdi Haresabadi, Kiana Amani, Zahra Jourahmad, Fabio Ferrarelli

**Affiliations:** 1 Department of Psychiatry, University of Pittsburgh, Pittsburgh, PA, United States; 2 Institute of Medical Science and Technology, Shahid Beheshti University, Tehran, Iran; 3 Department of Medical Physics and Biomedical Engineering, School of Medicine, Tehran University of Medical Sciences, Tehran, Iran; 4 School of Medicine, Tehran University of Medical Sciences, Tehran, Iran; 5 Addiction and Behavioral Sciences Research Center, North Khorasan University of Medical Sciences, Bojnurd, Iran; 6 Iranian Center of Neurological Research, Neuroscience Institute, Tehran University of Medical Sciences, Tehran, Iran; 7 Department of Neurosurgery, Baylor College of Medicine, Houston, TX, United States

**Keywords:** ALS, cortical onset theory, hyperexcitability, tFUS, TMS, upper motor neuron

## Abstract

Amyotrophic lateral sclerosis (ALS) is a devastating neurodegenerative disorder characterized by the progressive loss of upper motor neurons (UMNs) and lower motor neurons (LMNs). Despite significant advances in molecular and neuroimaging biomarkers, the initial site of pathology and the causal contribution of UMN dysfunction to disease progression remain undetermined. Accumulating neurophysiological evidence points to cortical hyperexcitability as an early and potentially upstream mechanism, raising the possibility that UMN pathology drives LMN degeneration through an anterograde dying-forward process. In this review, we synthesize findings from noninvasive brain stimulation (NIBS) studies, with particular emphasis on transcranial magnetic stimulation (TMS)-based neurophysiological markers of UMN dysfunction. We review evidence from TMS-electromyography (TMS-EMG) and TMS-electroencephalography (TMS-EEG) paradigms demonstrating cortical disinhibition and excitatory-inhibitory imbalance in ALS, consistent with impaired GABAergic interneuronal dysfunction and supportive of a cortical onset hypothesis. Finally, we propose integrating transcranial focused ultrasound (tFUS) with TMS as a novel experimental and translational framework to directly examine and modulate cortical hyperexcitability and test the causal role of UMN dysfunction in ALS. The combination of targeted neuromodulation with sensitive neurophysiological readouts in controlled experimental designs offers a promising avenue to advance mechanistic insight, refine biomarkers, and inform mechanism-based therapeutic strategies. Together, these approaches position noninvasive neurophysiology as a powerful tool for elucidating UMN dysfunction in ALS.

## 1 Introduction

Amyotrophic lateral sclerosis (ALS) is a devastating, rapidly progressive neurodegenerative disorder characterized by the degeneration of the motor nervous system resulting from dysfunction of both upper (UMNs) and lower motor neurons (LMNs) (1–4). Clinically, ALS most commonly begins with focal limb weakness and gradually spreads to involve bulbar and respiratory musculature, culminating in diffuse muscle atrophy, profound weakness, and ultimately respiratory failure (1, 5). The concurrent involvement of UMNs and LMNs is central to the clinical diagnosis of ALS and is fundamental to elucidating its underlying pathophysiology (6, 7).

Marked clinical and biological heterogeneity in ALS poses a major challenge to effective clinical trial design and therapeutic development. While clinical stratification can provide prognostic insight into disease progression and survival (8), existing assessment tools, most notably the Amyotrophic Lateral Sclerosis Functional Rating Scale-Revised (ALSFRS-R) (9, 10), lack the sensitivity required to capture subtle disease progression or therapeutic effects. This limitation highlights the urgent need for quantitative biomarkers that more directly reflect disease mechanisms and treatment response. Current biomarker approaches primarily include blood and cerebrospinal fluid (CSF) based measures, neuroimaging techniques such as MRI and PET, and quantitative metrics derived from applied neurophysiology, as reviewed in recent studies (10, 11). These biomarkers serve complementary roles in diagnosis, prognosis, patient stratification, and therapeutic monitoring. Diagnostic biomarkers differentiate ALS from phenotypic mimics, predictive and prognostic markers inform disease progression and survival, categorical biomarkers identify biologically distinct subgroups, and pharmacodynamic biomarkers provide evidence of target engagement, thereby enabling more efficient and shorter clinical trials (10). Despite these advances, the primary mechanisms underlying ALS onset and progression remain incompletely understood, and no single biomarker has yet achieved universal clinical adoption. Nonetheless, accumulating evidence supports their value as complementary tools within a multimodal biomarker framework.

In this review, we focus on neurophysiological biomarkers of ALS, with particular emphasis on noninvasive brain stimulation (NIBS) techniques such as transcranial magnetic stimulation (TMS) and emerging transcranial focused ultrasound (tFUS). We explore how these approaches can be used to interrogate UMN dysfunction and its potential role in disease initiation. Finally, we discuss how integrating these techniques may help bridge mechanistic insights with clinical application, advancing our understanding of ALS causality and therapeutic development.

## 2 ALS onset hypotheses

**Figure 1:**
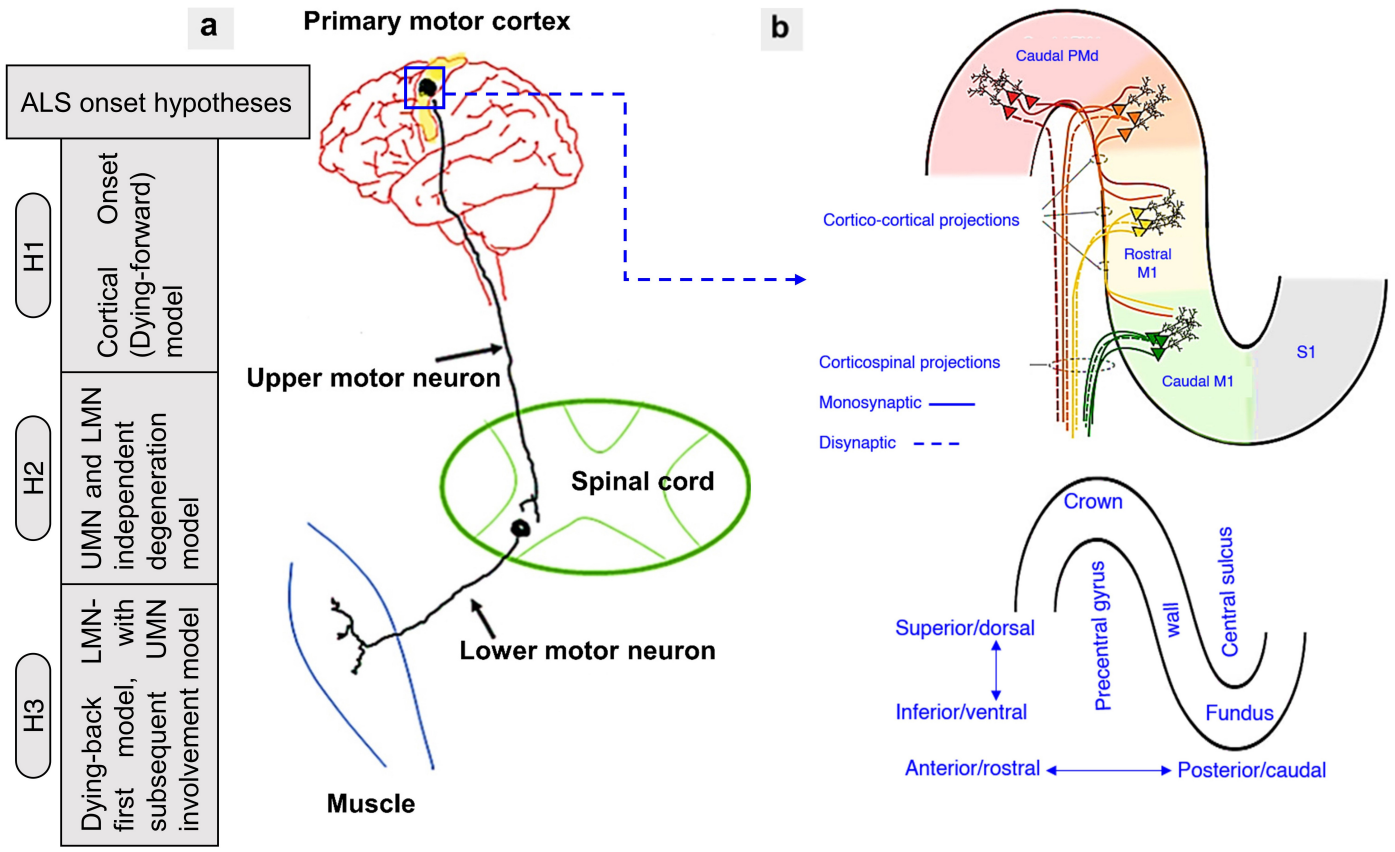
Overview of ALS onset hypotheses and the upper motor neurons (UMNs), lower motor neurons (LMNs) and the sensorimotor system. **(a)** UMNs arise in the cerebral cortex and descend through the corticospinal tract to synapse on LMNs in the spinal cord. LMNs, whose cell bodies lie in the anterior horn of the spinal cord and of motor cranial nerve nuclei, project via peripheral nerves to skeletal muscle (181). **(b)** Descending corticomotoneuronal pathways originating from the precentral gyrus contribute to motor evoked potentials (MEP) responses. Direct neuronal activation happen in the lip or rim of the motor hand knob region. Activation then spreads both rostrally and caudally within M1 via cortico-cortical synaptic transmission. **(a)** adapted and generated by courtesy of Fawzy Mahmoud Ali Abougalala (181) and panel **(b)** by courtesy of Aicee Dawn Calma et. al. (11) studies.

ALS is a neurodegenerative disease that affects both UMNs and LMNs (Figure 1a). LMNs, located in the spinal cord and brainstem, constitute the final common pathway for motor output to the limbs, face, larynx, and diaphragm. Functionally, they act as the circuit breakers of the nervous system: when LMNs degenerate, motor commands fail to reach the muscles, resulting in weakness, muscle wasting (amyotrophy), fasciculations, and muscle cramps.

UMNs originate in the motor cortex and brainstem and project through descending pathways to modulate LMNs, which in turn connect to muscles. Damage to UMNs leads to degeneration of the lateral corticospinal tracts, producing characteristic clinical features such as limb stiffness, exaggerated reflexes, and impaired voluntary motor control. The coordinated interaction between UMNs and LMNs collectively referred to as the corticomuscular system (Figure 1b), is essential for coordinated movement, and disruption of this coupling underlies the progressive motor impairment observed in ALS.

A fundamental and unresolved question in ALS research concerns the disease onset (11, 12). Three main, non-mutually exclusive hypotheses have been proposed (Figure 1a): (i) a cortical onset model (Dying-forward), in which degeneration of LMN is driven by excitotoxic anterograde signaling along descending corticomotoneuronal (i.e., a subset of corticospinal neurons that form direct connections with spinal motor neurons) pathways (13, 14); (ii) a model of parallel but interacting degeneration of UMNs and LMNs progressing in a contiguous or stochastic manner (15, 16); and (iii) a dying-back LMN-first model, with subsequent UMN involvement (17–20); Determining the causal relationship between UMN and LMN dysfunction is critical for elucidating the pathophysiology of ALS. In the following sections, we discuss how noninvasive neurophysiological approaches, including TMS and tFUS, can be used to directly probe and test these competing models of disease onset.

## 3 TMS and neurophysiological evidence in ALS

**Figure 2:**
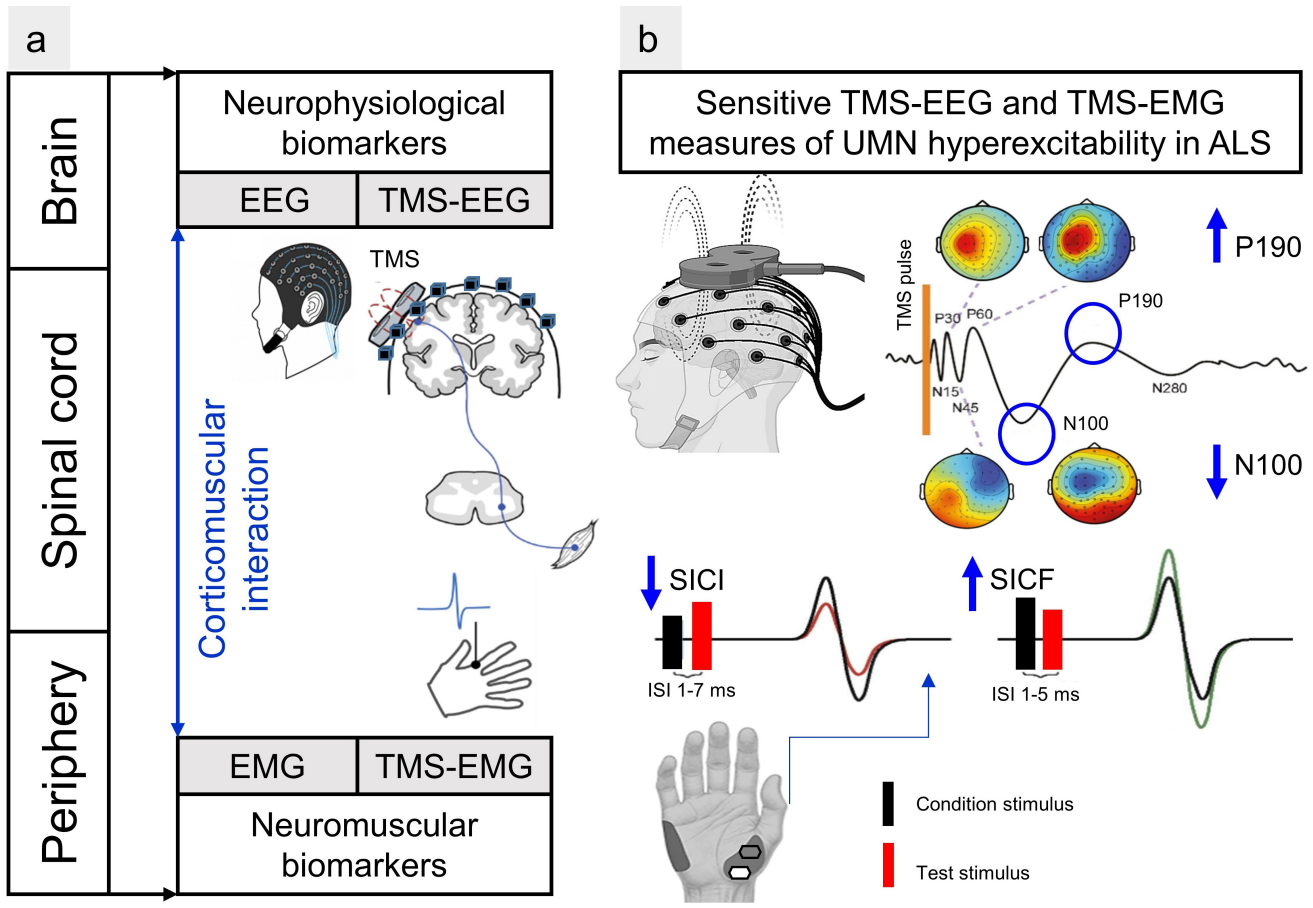
Corticomuscular interactions and sensitive neurophysiological markers of upper motor neuron (UMN) function in ALS. **(a)** illustrates corticomuscular interactions and the neurophysiological/neuromuscular modalities used to assess UMNs and LMNs function. **(b)** demonstrates sensitive UMNs markers derived from TMS-EEG and TMS-EMG that have been applied in ALS studies. Bold blue arrows show the direction of change for sensitive measures in ALS. SICI, short-interval intracortical inhibition; SICF, short-interval intracortical facilitation; ISI, interstimulus interval. Pictures used in **(b)**, including the TMS-EEG cap, TMS-evoked potentials (TEPs), and EMG responses, were adapted with courtesy of the study by Alberto Benussi and Steve Vucic (117).

TMS is a non-invasive, well-tolerated technique that enables *in vivo* assessment of UMN function in clinical and research settings (21–23). TMS methodological advances and the development of refined stimulation paradigms have substantially increased the sensitivity and clinical relevance of TMS for probing UMN physiology in ALS (10, 11). TMS delivers a brief, high-intensity current through a coil placed over the scalp, generating a rapidly changing magnetic field that reaches peak strengths of approximately 2 Tesla and is oriented perpendicular to the coil surface (21, 24). This magnetic field induces an electric field within the motor cortex (Figure 2), primarily activating axons of excitatory corticocortical neurons that trans-synaptically depolarize corticospinal pyramidal neurons, with concomitant recruitment of GABAergic interneuronal networks that modulate the excitatory-inhibitory balance of the evoked response (11, 24, 25). At higher stimulation intensities, direct activation of corticospinal axons may also occur (Figure 1b).

The resulting corticospinal output is measured as a motor-evoked potential (MEP) recorded from a target muscle, reflecting depolarization of spinal motor neurons via descending corticospinal pathways (Figure 2). The neuronal populations recruited by TMS depend on multiple parameters, including coil geometry and penetration depth, orientation of the induced current, pulse waveform, stimulation intensity, and the temporal pattern of stimulation (24, 26–30). We systematically reviewed the TMS literature in ALS across three electronic databases: PubMed, Scopus, and Web of Science. The following search strategies were applied: PubMed: [“Amyotrophic Lateral Sclerosis” (Title/Abstract) OR “Amyotrophic Lateral Sclerosis” (MeSH) OR ALS (Title/Abstract)] AND [“Transcranial Magnetic Stimulation” (Title/Abstract) OR “Transcranial Magnetic Stimulation” (MeSH) OR TMS (Title/Abstract)]. Scopus: (“Amyotrophic Lateral Sclerosis” OR ALS) AND (“Transcranial Magnetic Stimulation” OR TMS). Web of Science: AB = (“Amyotrophic Lateral Sclerosis” OR ALS) AND (“Transcranial Magnetic Stimulation” OR TMS) OR TI = (“Amyotrophic Lateral Sclerosis” OR ALS) AND (“Transcranial Magnetic Stimulation” OR TMS).

Studies were included if they were published as peer-reviewed journal articles, were written in English, involved human participants with ALS or motor neuron disease, and reported TMS-based neurophysiological outcomes. Study-level details of the identified articles are summarized in Table 1 and Supplementary Table 1. Collectively, this body of work establishes TMS as a robust neurophysiological tool for assessing UMNs dysfunction in ALS, providing mechanistic insights into disease pathophysiology and supporting the development of diagnostic and therapeutic-response biomarkers (11, 25, 31, 32).

**Table 1:** Transcranial magnetic stimulation (TMS)-electroencephalography (EEG)/electromyography (EMG) measures in amyotrophic lateral sclerosis (ALS).

TMS EMG/EEG parameter	Physiological mechanism	Circuitry and neurochemical basis	Clinical relevance in ALS
Motor threshold (RMT)	The minimum magnetic stimulation intensity required to elicit a motor response	Reflects overall motor cortex excitability and corticospinal integrity; influenced by membrane excitability, synaptic drive, and motor pathway integrity	Often decreased early in ALS due to cortical hyperexcitability, then increase later with neuronal loss
Motor evoked potential (MEP)	The evoked motor response produced by TMS over motor cortex	Reflects the combined excitability of cortical, corticospinal, spinal, and peripheral motor pathways	Variable depending on disease stage and pathway integrity
Central motor conduction Time (CMCT)	The latency from cortical stimulation to muscle response attributable to central motor pathways	Measures conduction along the fastest corticomotoneuronal fibers and their synaptic transmission	Prolongation suggests corticospinal tract dysfunction and upper motor neuron involvement
Cortical silent period (CSP)	A period of EMG suppression following an MEP during voluntary contraction	The later part is thought to depend largely on GABA_B_ mediated inhibition, though stimulus intensity and other circuit properties also contribute	Often shortened in ALS, consistent with reduced cortical inhibition, but findings can vary by method and disease stage
Short-interval intracortical inhibition (SICI)	Inhibition of a test MEP by a preceding subthreshold conditioning stimulus at short intervals	Mediated mainly by inhibitory intracortical interneurons acting through GABA_A_ receptors	One of the most consistent abnormalities in ALS; often present early and may precede overt lower motor neuron signs
Long-interval intracortical inhibition (LICI)	Suppression of a test MEP by a preceding suprathreshold conditioning stimulus at interstimulus intervals of ~50–300 ms	Mediated by long-latency inhibitory interneuronal populations acting through postsynaptic GABA_B_ receptors	LICI reduction has been reported in ALS and is associated with greater disease severity, suggesting degeneration of long-latency inhibitory circuits. This finding needs to be replicated in more studies
Short-interval intracortical facilitation (SICF)	Facilitation of an MEP by a second stimulus at short interstimulus intervals	Reflects facilitatory intracortical interactions and likely cortico-cortical or axonal synchronization mechanisms	Increased in ALS, suggesting imbalance between excitation and inhibition, but findings are less uniform than for SICI
Intracortical facilitation (ICF)	Facilitation of an MEP at longer interstimulus intervals	Reflects facilitatory intracortical circuits, likely involving glutamatergic mechanisms, but its exact physiology is less settled than SICI	Findings are variable in ALS and are generally less consistent than SICI
Triple stimulation technique (TST)	A collision-based method used to estimate the proportion of activated corticomotoneurons	Accounts for MEP dispersion and cancellation to provide a more precise estimate of corticospinal output	Reduced values indicate corticospinal dysfunction and can reveal subclinical upper motor neuron involvement
N100 TEP component	A negative TMS-evoked EEG component occurring approximately 100 ms after stimulation	Commonly associated with cortical inhibitory processes, but it is not a specific direct marker of GABA_B_ activity	Deceased in ALS and can provide evidence of abnormal cortical inhibition, but interpretation should remain cautious
P190 TEP component	A positive TMS-evoked EEG component occurring approximately 180–190 ms after stimulation	A later TEP component influenced by broader cortical and non-cortical processes; its exact physiological meaning is less specific	Increased in ALS, but it should not be interpreted as a definitive marker of cortical hyperexcitability on its own

Using single-, paired-, and triplestimulation technique TMS paradigms, as well as electroencephalography (EEG) and combined EEG-Electromyography (EMG) approaches, prior studies have identified multiple physiological biomarkers of UMNs dysfunction in ALS (10, 11). In addition, our group and others have demonstrated that EEG and concurrent EEG-EMG recordings at rest as well as during static and dynamic muscle contractions can be used to characterize cortical hyperexcitability (33) and that alterations in corticomuscular delay and strength-related metrics may serve as candidate biomarkers of UMN dysfunction (34–41). Building on these findings, the present review focuses on sensitive TMS-EEG and TMS-EMG metrics providing direct evidence for top-down alterations in corticomuscular interactions, consistent with the cortical onset model of ALS. We further evaluate the clinical applicability of these metrics for elucidating mechanisms of UMN hyperexcitability in ALS.

### 3.1 TMS-EMG measures in ALS

Single pulse TMS (spTMS): spTMS is a non-invasive neurophysiological technique that uses a brief, high-intensity magnetic field to induce an electrical current in the primary motor cortex. This focal stimulation depolarizes corticospinal neurons, resulting in a MEP recorded via electromyography (EMG) from a target muscle. By quantifying MEP characteristics and the stimulus intensity required to elicit them, spTMS provides a window into the excitability of the human motor system and the integrity of descending corticospinal pathways with the following outcome measures (42).

Resting motor threshold (RMT): RMT is a fundamental marker of corticomotoneuronal excitability and reflects the function of voltage-gated sodium channels and glutamatergic neurotransmission. It is conventionally defined as the lowest stimulation intensity capable of eliciting an MEP ≥50 μV in 50% of at least 10 trials (11, 25, 42, 43). In ALS, RMT findings are heterogeneous. Reduced RMT has been reported in early disease stages, particularly contralateral to the site of symptom onset, often preceding muscle wasting and correlating with hyper-reflexia and fasciculations, consistent with corticomotoneuronal hyperexcitability (25, 44–46). As the disease progresses and the corticomotoneuronal pool is degenerating, RMT typically increases (47–54), and some patients ultimately demonstrate cortical inexcitability (44). This suggests that using TMS in early stages of the disease would be more beneficial. Additionally, RMT appears to be influenced by racial background, with higher thresholds observed in Japanese ALS cohorts (11, 55, 56). Taken together, the variability of RMT across disease stages and populations limits its utility as a standalone diagnostic biomarker in ALS.

MEP amplitude and stimulus-response (SR) curve: MEP amplitude and the SR curve probe a population of corticomotoneurons distinct from those indexed by RMT. A steeper SR slope is indicative of increased glutamatergic drive and/or reduced GABAergic inhibition and has been proposed as a marker of cortical hyperexcitability in ALS (25, 57, 58). To account for peripheral nerve variability and LMN degeneration, the MEP/CMAP (compound muscle action potential) ratio is often utilized. This normalization provides a more robust measure of central motor integrity (59–61). Increased MEP amplitudes and MEP/CMAP ratios are frequently observed early in ALS, help distinguish ALS from mimic disorders, and support the presence of widespread corticomotor hyperexcitability (46, 62–64).

Cortical Silent Period (CSP): The CSP refers to the transient interruption of voluntary EMG activity following a suprathreshold TMS pulse during muscle contraction (65). CSP duration is defined as the interval from MEP onset to the resumption of ongoing EMG activity (65, 66). CSP primarily reflects GABA_B_ receptor-mediated cortical inhibition, with additional contributions from spinal inhibitory mechanisms (27, 67–69). In ALS, a shortened or absent CSP is a common early finding, reflecting impairment of long-latency GABAergic inhibitory circuits (70–75). However, despite its pathophysiological relevance, CSP has shown only modest diagnostic utility (11, 31, 55, 76).

Central Motor Conduction Time (CMCT): CMCT measures the time required for neural impulses to travel from the motor cortex to spinal motor neurons (10, 11, 77). Although CMCT prolongation reflects corticospinal tract dysfunction and may reveal subclinical UMN involvement in ALS, its diagnostic value is limited by inconsistent correlations with clinical UMN signs and rating scales (55, 61, 70, 76).

Paired-pulse (pp) TMS: ppTMS in which a conditioning stimulus precedes a test stimulus at specific interstimulus intervals (ISIs) is considered the gold standard for interrogating the intracortical interneuronal dysfunction central to ALS pathophysiology (Figure 2b) (22, 25, 27, 55). Paradigms commonly applied in ALS include short-interval intracortical inhibition (SICI), short-interval intracortical facilitation (SICF), intracortical facilitation (ICF), and long-interval intracortical inhibition (LICI), with SICI demonstrating the greatest diagnostic relevance (22, 25, 27, 55).

Short-interval intracortical inhibition (SICI): SICI is a sensitive marker of motor cortical inhibition mediated by GABA_A_ receptors (58). It is elicited when a suprathreshold test stimulus (TS) is preceded by a subthreshold conditioning stimulus (CS) at interstimulus intervals (ISIs) of 1–7 ms, typically using the threshold-tracking paired-pulse TMS (TT-TMS) approach (Figure 2b). In TT-TMS, TS intensity is continuously adjusted to maintain a target MEP amplitude of 0.2 mV (± 20%) across ISIs (11, 55, 58, 66, 78, 79). A reduction or absence of SICI is one of the most consistent neurophysiological abnormalities in ALS, irrespective of the methodology (31, 46, 56, 63, 80–85). Importantly, SICI reduction often precedes LMN signs and correlates with disease progression and shorter survival (31, 63, 64).

Long interval intracortical inhibition (LICI): LICI refers to the suppression of a test MEP elicited by a suprathreshold conditioning stimulus delivered at interstimulus intervals of 50–300 ms (66, 86, 87). This effect is thought to be mediated by long-latency inhibitory interneuronal populations acting through postsynaptic GABA_B_ receptors (58, 88–90). Reduced LICI has been reported in ALS and is associated with greater disease severity (91, 92), suggesting degeneration of long-latency inhibitory circuits, although further confirmation is needed (11, 58).

Short interval intracortical facilitation (SICF): SICF indexes high-threshold facilitatory interneuronal circuits and is elicited by delivering a conditioning stimulus at threshold or suprathreshold intensity 1–5 ms before a test stimulus (Figure 2b). Alterations in SICF have been reported in ALS and are thought to contribute to disease pathogenesis (93–96).

Intracortical facilitation (ICF): ICF reflects activity of longer-latency facilitatory circuits with lower activation thresholds than those mediating SICF and is elicited at ISI of 8–30 ms (66, 79). Although its physiological basis is not fully understood, ICF likely involves low-threshold glutamatergic excitatory cortical circuits, with possible spinal contributions (11, 26, 66, 96–98). In ALS, increased SICF is commonly observed, whereas ICF findings are more variable (22, 25, 80, 99–101). Elevated SICF often co-occur with reduced SICI (11, 99), reflecting an imbalance between cortical excitation and inhibition. This imbalance has been quantified using the index of excitation (IE), which is increased in ALS and correlates with greater functional impairment and more pronounced UMN signs. While SICF alone requires further validation as a diagnostic marker, its combination with SICI reduction shows promise as a sensitive neurophysiological biomarker (11, 99, 102).

Triple stimulation technique (TST): the TST was developed to overcome the variability and desynchronization inherent to conventional MEPs elicited by spTMS. Using a collision paradigm involving three sequential stimuli (cortical, distal peripheral, and proximal peripheral), TST provides a precise estimate of the proportion of activated corticomotoneurons (11, 59, 103–105). A suprathreshold TMS pulse is delivered first to the motor cortex, followed by a distal peripheral nerve stimulus and a proximal peripheral stimulus at timed intervals. The descending corticospinal volley collides with the antidromic impulse generated by the second stimulus, resulting in phase cancellation, while the third stimulus evokes a supramaximal compound muscle action potential (CMAP). Comparison of CMAP amplitude and area between conditioned and unconditioned conditioned TST yields ratios reflecting the proportion of activated corticomotoneurons (106–108).

Reduced TST CMAP amplitude ratios have been reported in more than 60% of ALS patients (11, 109), and demonstrate greater sensitivity than MEP amplitude reduction or CMCT prolongation (109). These reductions correlate with functional disability and UMN impairment and can detect subclinical UMN dysfunction, supporting its diagnostic relevance (110–113). With the increasing availability of commercial TST software, clinical implementation as an adjunct diagnostic tool may become feasible in the future.

### 3.2 TMS-EEG measures in ALS

The combination of transcranial magnetic stimulation with electroencephalography (TMS-EEG) enables non-invasive assessment of cortical excitability with millisecond temporal resolution through the measurement of transcranial evoked potentials (TEPs). TEPs are characterized by reproducible spatio-temporal sequences of positive and negative peaks, and TMS-EEG has attracted growing interest across a range of research and clinical applications (Figure 2b) (114, 115). In ALS, however, the application of TMS-EEG is relatively recent, with only one study published to date (116), as most prior work has relied on TMS-EMG rather than direct cortical measures (117).

In this seminal TMS-EEG study, single-pulse and a novel inhibitory paired-pulse paradigm were applied in 21 individuals with ALS compared with healthy controls. ALS patients exhibited marked alterations in TEP morphology, including a reduced N100 and an increased P190 component; findings interpreted as reflecting GABAergic dysfunction and cortical disinhibition. Furthermore, the paired-pulse TMS-EEG paradigm demonstrated reduced short-interval intracortical inhibition of the N100, which correlated with longer disease duration and greater functional disability. Relatedly, the pharmacological basis of the TEP components is well established through pharmaco-TMS-EEG challenge studies. Specifically, the N100 component reflects GABA_B_ receptor-mediated cortical inhibition, as it is increased by baclofen, a GABA_B_ agonist (118), as well as the local GABA/glutamate balance (119, 120). Furthermore, the late positive TMS-evoked component around 180–190 ms (P180/P190) is often co-modulated with the N100 and has been interpreted as part of the late inhibitory response to cortical stimulation (121). More broadly, altered TMS-based inhibitory measures and TEP abnormalities have been reported across neurodegenerative conditions, including Alzheimer's disease, Parkinson's disease, and non-Alzheimer's dementias, supporting the general utility of TMS-EEG as a probe of cortical dysfunction (115, 122–125). However, since late TEPs can be partially influenced by auditory responses to the TMS coil click, rigorous masking and sham-control procedures remain important in future studies (114). Combined, these findings provide the first direct EEG-based evidence of cortical interneuron circuit dysfunction in ALS and support cortical hyperexcitability as a core pathophysiological feature of the disease (116). However, this interpretation remains provisional since TMS-EEG evidence in ALS is still limited and requires replication in larger cohorts.

Collectively, aforementioned findings support the cortical onset hypothesis of ALS and motivate further causal interrogation of cortical hyperexcitability. One promising avenue to achieve this is the integration of emerging neuromodulation technologies, particularly combined tFUS-TMS paradigms, as outlined below.

## 4 Unraveling the UMN information in ALS using combined tFUS-TMS paradigm

Transcranial focused ultrasound (tFUS) has recently emerged as a powerful non-invasive neuromodulation technique with the unique ability to modulate neural activity and functional connectomes with high spatial precision and depth penetration (126–130). Unlike conventional non-invasive brain stimulation methods, tFUS can target both superficial and deep brain structures with millimeter-scale resolution using ultrasound waves. The key parameters defining a tFUS protocol can be understood intuitively by considering how energy is delivered over time. The fundamental frequency (typically ~200–700 kHz) refers to how fast the ultrasound waves oscillate, analogous to the pitch of a sound, and influences how well the waves penetrate the skull and interact with tissue. The pulse repetition frequency (PRF) describes how often pulses are delivered per second, similar to the rhythm of tapping. The duty cycle indicates the proportion of time the ultrasound is “on” vs. “off” within each pulse cycle, effectively controlling how continuous or intermittent the stimulation is. The sonication duration reflects how long a stimulation train lasts, while acoustic intensity (e.g., spatial-peak temporal-average intensity, I_SPTA_) represents the strength of the energy delivered to the tissue. Additional parameters further refine the temporal pattern of stimulation. The burst length determines how long each individual pulse lasts, while the inter-stimulus interval specifies the pause between successive stimulation trains. The total number of pulses defines the cumulative exposure over a session. Together, these parameters shape both the amount and timing of energy delivered to the brain. For more details on other parameters, how tFUS works, the essential components of the system, and how the experiment could be conducted, please see (126).

**Figure 3:**
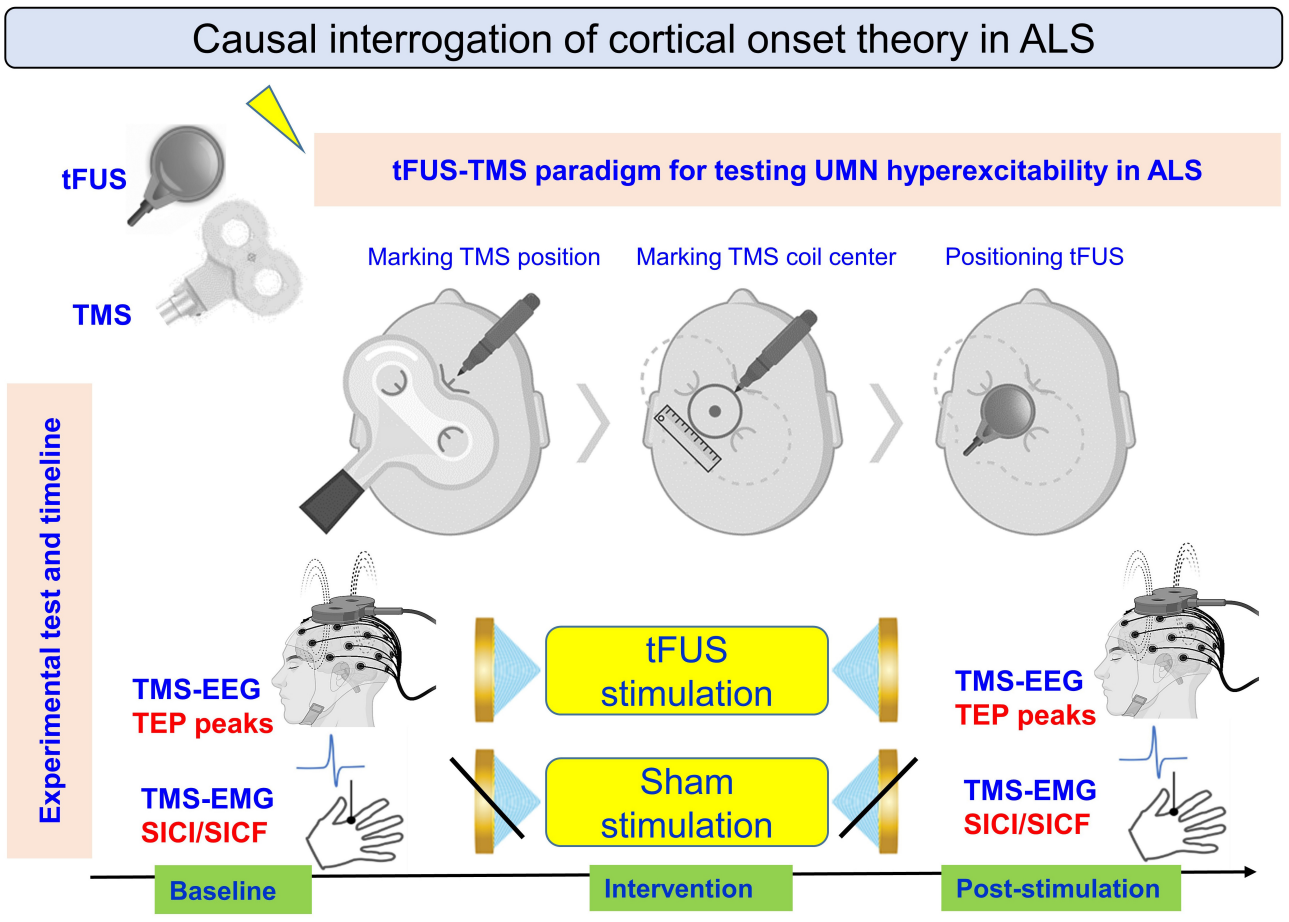
Causal interrogation of the cortical onset theory in ALS: experimental design using a combined tFUS-TMS paradigm to assess upper motor neuron dysfunction. Cortical hyperexcitability may be utilized as a modifiable pathophysiology biomarker of the cortical onset hypothesis in ALS. Specifically, TMS-evoked EEG potentials (TEPs) and TMS-MEP parameters, including MEP amplitude, SICI, and SICF could be obtained from the primary muscle of interest before and after the application of an inhibitory tFUS protocol in a double-blind experimental design. The tFUS transducer is positioned on the center of the TMS coil, placed over the primary motor cortex hotspot. Pictures including the TMS-EEG cap, and transducer position on the head were adapted with courtesy of Po-Yu Fong et al. (117), and Alberto Benussi and Steve Vucic (117).

Depending on stimulation parameters, tFUS can induce either excitatory or inhibitory effects, making it a versatile tool for probing neural circuits and testing causal mechanisms underlying neurophysiological dysfunction (126, 131–133). Within this context, “excitatory” and “inhibitory” tFUS refer to sonication protocols that are hypothesized to increase or decrease neuronal excitability, respectively. Excitatory protocols typically utilize higher duty cycles (30% to 50%) paired with a higher PRF (often > 1 kHz) and short sonication bursts (200–500 ms) to drive rapid membrane depolarization via mechanosensitive ion channels, resulting in increased local field potentials, elevated BOLD fMRI signals, and increased glutamate release (126, 132–135). Clinically, these excitatory protocols are leveraged for neurorehabilitation, such as facilitating motor recovery post-stroke or enhancing cognitive function (136). In contrast, inhibitory protocols deploy lower duty cycles (5% to 10%), lower PRFs (10 Hz to 250 Hz), and longer, sustained or intermittent sonication durations (10–60 s) to suppress ongoing neural activity, a mechanism driven by the activation of GABAergic pathways or minor physiological micro-heating. These inhibitory paradigms manifest as reduced MEP amplitudes and suppressed BOLD signals, offering therapeutic utility for down-regulating hyperexcitable brain states found in a variety of disorders (126, 132–135). This characteristic is particularly relevant in ALS, where cortical hyperexcitability is supported as a central pathophysiological marker (11, 22, 66, 116, 117). Combining tFUS with sensitive TMS-based outcome measures therefore offer a powerful experimental and translational framework. Well-established TMS metrics, such as SICI, its complementary SICF, as well as other sensitive TMS-EEG and TMS-EMG indices, can be leveraged to quantify the effects of targeted cortical perturbation (Figure 3). Of note, growing evidence underscores the particular value of TMS-EEG measures for capturing interneuron excitability and cortical inhibitory dynamics, underscoring their importance for mechanistic investigations in ALS (116, 117).

Within this framework, the combination of inhibitory tFUS protocols (i.e., with the goal to suppress hyperexcitability), with pre- and post-tFUS assessments with sensitive TMS-EMG and TMS-EEG measures, may crucially contribute to directly implicate cortical dysfunction in ALS pathogenesis (Figure 3). When embedded within double-blind, controlled experimental or clinical trial designs and combined with clinical and behavioral outcome measures, this approach has the potential to clarify disease mechanisms, refine biomarkers, and inform therapeutic strategies aimed at reducing motor cortical hyperexcitability and potentially slowing disease progression.

More broadly, the ability of tFUS to modulate both cortical and subcortical circuits, as well as corticospinal and corticomuscular pathways, provides a unique opportunity to interrogate causal interactions across multiple levels of the sensorimotor system (Figure 1). By integrating these complementary neuromodulation and neurophysiological techniques, future studies may yield deeper mechanistic insight into upper motor neuron dysfunction in ALS and support the development of mechanism-based interventions.

Importantly, this translational framework is already reflected in ongoing clinical trial efforts (https://clinicaltrials.gov/study/NCT06681610), which aim to determine whether inhibitory neuromodulation alters motor cortical SICI over 8 weeks (Stage 1) and whether such changes translate into clinically meaningful effects on ALSFRS-R scores over 6 months (Stage 2). These efforts further underscore the promise of combined tFUS-TMS approaches for advancing ALS research and therapeutic development.

## 5 Discussion

In this review, we examined how advances in neurophysiological technologies are reshaping the understanding, diagnosis, and pathophysiology of ALS. While imaging and fluid-based biomarkers have driven important progress in recent years (10), their clinical applicability is often constrained by cost, invasiveness, and limited sensitivity to dynamic brain function. In contrast, neurophysiological approaches, particularly TMS and its integration with EMG and/or EEG (TMS-EMG and TMS-EEG, Figure 2), provide a unique, non-invasive window into corticomotoneuronal function, cortical excitability, and plasticity with millisecond-level temporal resolution (117). We further highlighted that the translational potential of combining TMS with emerging technological techniques such as tFUS, which may accelerate the transition of these methods from research settings into clinical applications (Figure 3). By detecting subtle network-level dysfunctions that may precede overt structural degeneration, these techniques hold promise for identifying ALS at prodromal or even presymptomatic stages, when therapeutic interventions may be most effective.

Neurophysiological biomarkers of UMNs dysfunction provide critical insight into ALS pathogenesis. In particular, corticomotoneuronal hyperexcitability has been proposed as a key pathogenic mechanism, potentially driving LMN degeneration through an anterograde glutamatergic process commonly referred to as the dying-forward hypothesis (11, 137). Converging evidence from TMS-EMG studies, especially those employing threshold-tracking paradigms, provides strong support for the cortical onset (dying-forward) hypothesis (11, 55). Reduced SICI, a hallmark of cortical disinhibition, correlates with biomarkers of LMN dysfunction and degeneration (64, 83, 138), precede LMN loss (11, 83, 139), and is associated with distinct clinical phenotypes and trajectories of disease progression (46, 140–142). Moreover, cortical hyperexcitability is consistently linked to faster disease progression, greater functional impairment, and reduced survival (100, 143, 144). In parallel, increased activity of high-threshold facilitatory interneuronal networks, reflected by increased SICF, has been observed in ALS and correlates with greater UMN dysfunction and functional decline (31). Importantly, the presence of cortical hyperexcitability reliably distinguishes ALS from neuromuscular mimic disorders, arguing against the interpretation of these changes as a compensatory plasticity phenomenon (11, 31).

Cortical hyperexcitability may therefore constitute a critical upstream event that within the multistep pathogenic framework of ALS preceding LMN degeneration (145–147). Nonetheless, ALS is a highly complex neurodegenerative disorder arising from the interplay of genetic, molecular, and environmental factors. Multiple molecular mechanisms have been implicated, including glutamate-mediated excitotoxicity, oxidative stress, impaired autophagy, dysregulated RNA and lipid metabolism, mitochondrial dysfunction, defective axonal transport, aberrant neuroinflammatory responses, and proteasomal impairment (1, 11, 145, 148). In addition, protein aggregation driven by specific ALS-associated genetic mutations further contributes to neuronal vulnerability (11). Evidence from rodent models indicates that cortical hyperexcitability arises from altered excitability of both excitatory and inhibitory cortical neurons and temporally precedes UMN structural degeneration (11, 149, 150). Within the multistep model of ALS, cortical hyperexcitability may therefore represent an upstream pathophysiological event that precedes and potentially drives LMN degeneration through a dying-forward mechanism, rather than simply reflecting established UMN loss. Whether it constitutes a primary initiating trigger or emerges at a later stage of the pathogenic cascade remains to be determined (11, 149, 150). Importantly, corticomotoneuronal hyperexcitability may also serve as a conduit for the clinical heterogeneity observed in ALS. The frequently reported contiguous spread of disease, with concordance between UMN and LMN dysfunction at the onset site, may be explained by regionally higher corticospinal tract innervation density and correspondingly greater local corticomotoneuronal hyperexcitability. This is supported by TMS studies showing focal cortical hyperexcitability at the disease onset site, and by transgenic mouse models demonstrating that TDP-43 mis-localization to UMNs drives spinal motor neuron degeneration through anterograde excitotoxic signaling (151). That said, whether cortical hyperexcitability represents an initiating pathogenic trigger or emerges as a downstream consequence during later stages of the ALS multistep process remains to be determined (11, 145).

Notably, a recent TMS-EEG study directly interrogated cortical interneuronal function in ALS (116). This work demonstrated localized dysfunction of GABAergic inhibitory circuits, providing initial evidence that cortical hyperexcitability in ALS arises from cortical disinhibition independent of LMNs pathology. Importantly, GABAergic dysfunctions correlated with greater clinical disability and longer disease duration, underscoring their pathophysiological relevance. Together, these findings further support the cortical onset hypothesis and identify cortical hyperexcitability as a mechanistically meaningful ALS feature that warrants causal testing in future studies (Figure 3).

From a diagnostic perspective, the absence of a pathognomonic test makes ALS diagnosis inherently challenging. Current diagnostic practices rely on identifying progressive UMNs and LMNs signs while excluding potential mimicking disorders (4, 7, 10, 11, 148, 152). Several clinical and neurophysiological diagnostic criteria have been developed to facilitate earlier and more definitive diagnosis (6, 10, 11, 152–154). However, the revised El Escorial and Awaji-Shima criteria are complex and incorporate multiple levels of diagnostic certainty (definite, probable, and possible ALS) (6, 152–154). This complexity, coupled with a heavy reliance on clinical assessment of UMNs dysfunction, has resulted in limited sensitivity and poor inter-rater reliability (11, 155–158). The more recent Gold Coast criteria simplify diagnosis by eliminating levels of certainty and defining ALS based on the presence of UMNs and LMNs dysfunction in one body region, or LMNs dysfunction in two regions, in the context of disease progression (7). While the Gold Coast criteria have demonstrated improved sensitivity, challenges in the objective assessment of UMNs dysfunction persist (7, 159).

In this context, the incorporation of objective neurophysiological biomarkers, such as SICI, SICF, and TEP components, may substantially enhance diagnostic accuracy. Additionally, the triple stimulation technique, including measurements of TST amplitude and area ratios, offers further diagnostic value, with reported sensitivities ranging from 54 to 100% (110–113). Importantly, TST may also aid in differentiating ALS from mimicking disorders by detecting proximal conduction block (110, 160–163).

Taken together, and in light of the growing consensus around TMS-based paradigms, we suggest that future ALS clinical trials consider incorporating measures of SICI, SICF, and/or TEPs, as well as TST metrics where feasible, while considering the causal interrogation of cortical hyperexcitability using a combined TMS and tFUS approach. Of note, we propose tFUS here due to its applicability for both superficial and deep brain targeting with high spatial resolution; however, other NIBS approaches, such as TMS protocols [e.g., include low-frequency rTMS (typically 1 Hz) and continuous theta-burst stimulation (cTBS)] (164), may also be used in cases of UMN interrogation where coarser spatial targeting is sufficient. At the same time, it is essential to acknowledge that additional validation is required before these techniques can be fully integrated into routine clinical practice, as discussed below.

### 5.1 Limitations

Despite extensive application of non-invasive brain stimulation across neurological populations, the clinical translation of TMS, TMS-EMG, and TMS-EEG in ALS remains at an early stage (115, 116, 165–175). Most studies to date have been conducted in a limited number of highly specialized centers, restricting generalizability (11). The need for specialized equipment, software, and operator expertise has hindered widespread adoption, especially for TMS-EEG (176), and has limited systematic assessments of inter-rater and intra-rater reliability, variability, and cross-center standardization. Notably, only a single TMS-EEG study in ALS has been published to date (116), underscoring the nascent state of this approach.

Cortical excitability is stage dependent in ALS, and these stage-dependent changes suggest that TMS is likely to be most informative in the early phases of ALS, when cortical hyperexcitability can still be reliably detected. Accordingly, as disease advances and motor neuron degeneration becomes more pronounced, the utility of TMS may be reduced, particularly in very late-stages of the disease when cortical responses may be absent. Therefore, interrogating upper motor neuron dysfunction early in the disease course may provide greater diagnostic and prognostic value.

Threshold-tracking TMS remains available only in select expert laboratories, limiting translatability and the establishment of normative reference values. The absence of standardized acquisition and analysis protocols across centers further constrains reproducibility and large-scale implementation. Encouragingly, recent commercialization of off-the-shelf TMS-EEG systems and emerging advanced technologies such as tFUS (126), offers a promising opportunity to expand accessibility, scalability, and multicenter validation.

Finally, it is important to emphasize that ALS is a highly complex, multi-step neurodegenerative disorder (145–147). While most neurophysiological evidence derives from the sensorimotor system, accumulating data indicates a more widespread cortical dysfunction in ALS (11, 116). Beyond cortical targets, ALS involves subcortical, brainstem, and cerebellar structures (177, 178). Subcortical degeneration has been reported in the thalamus, caudate, pallidum, hippocampus, and nucleus accumbens. Brainstem pathology includes local atrophy and white-matter degeneration in corticospinal and fronto-pontine tracts, while cerebellar abnormalities have been linked to motor and extra-motor phenotypes (178), suggesting that these deep regions may represent circuit-level targets for tFUS. For example, stimulation of thalamocortical or cerebellothalamic pathways could be used to modulate cortical excitability, while tFUS of basal ganglia circuits could affect motor initiation and network synchronization. The ability of tFUS to selectively engage deep nodes within these networks may provide both mechanistic insight and therapeutic potential. At the same time, a comprehensive understanding of cortical hyperexcitability will require a broader network-level perspective, encompassing transcallosal interactions, large-scale brain networks (179), and the contribution of brainstem circuits (178) and the cerebellum (180). In this regard, combined neuromodulation approaches, such as integrated tFUS-TMS paradigms (Figure 3) may offer a feasible strategy to interrogate cortical and subcortical circuits while simultaneously measuring sensitive neurophysiological biomarkers (e.g., TMS-EMG and/or TMS-EEG indices) longitudinally.

## 6 Conclusions

Converging evidence from neurophysiological markers of UMNs dysfunction supports the cortical onset theory of ALS, with reduced SICI and increased SICF emerging as complementary and reproducible measures of cortical hyperexcitability. These findings highlight cortical hyperexcitability as a core pathophysiological feature of ALS and underscore the need for further causal mechanistic investigation using translational approaches such as combined tFUS-TMS paradigms. Given the limitations of current clinical outcome measures, incorporating objective neurophysiology-based biomarkers, including TMS-EMG and/or TMS-EEG metrics, as primary endpoints in future ALS clinical trials may improve sensitivity, enhance mechanistic insight, and potentially accelerate therapeutic development.
